# Excitation-Inhibition Balanced Neural Networks for Fast Signal Detection

**DOI:** 10.3389/fncom.2020.00079

**Published:** 2020-09-03

**Authors:** Gengshuo Tian, Shangyang Li, Tiejun Huang, Si Wu

**Affiliations:** ^1^School of Electronics Engineering and Computer Science, Peking University, Beijing, China; ^2^IDG/McGovern Institute for Brain Research, Peking-Tsinghua Center for Life Sciences, Academy for Advanced Interdisciplinary Studies, Peking University, Beijing, China

**Keywords:** E-I balanced network, optimal noise structure, Fokker-Planck equation, fast tracking, asynchronous state

## Abstract

Excitation-inhibition (E-I) balanced neural networks are a classic model for modeling neural activities and functions in the cortex. The present study investigates the potential application of E-I balanced neural networks for fast signal detection in brain-inspired computation. We first theoretically analyze the response property of an E-I balanced network, and find that the asynchronous firing state of the network generates an optimal noise structure enabling the network to track input changes rapidly. We then extend the homogeneous connectivity of an E-I balanced neural network to include local neuronal connections, so that the network can still achieve fast response and meanwhile maintain spatial information in the face of spatially heterogeneous signal. Finally, we carry out simulations to demonstrate that our model works well.

## 1. Introduction

To survive in natural environments, animals have developed, through millions of years evolution, the ability to process sensory inputs rapidly. For instance, studies have shown that human subjects can perform complex visual analyses within 150 ms (Thorpe et al., 1996), and the response latency of neurons in the visual cortex of monkeys is as short as tens of milliseconds (Raiguel et al., 1999; Sugase et al., 1999).

Meanwhile, many artificial engineering systems have high demands for real-time processing of rapidly varying signals. This is exemplified by the recently developed Spike Camera (Dong et al., 2017), which has a sampling rate of up to 40, 000 frames per second (fps), far surpassing conventional cameras' 60 fps. This allows it to capture high-speed objects and their textual details, which can be used on real-time motion detection, tracking, and recognition if we have the appropriate algorithms and computing platforms. However, the processing speed of traditional algorithms often cannot meet such demands.

The balance of excitation and inhibition is a general property of neural systems. The excitation-inhibition (E-I) balanced neural network was first proposed to explain the irregular firing of cortical neurons widely observed in the cortex (Softky and Koch, 1993; Shadlen and Newsome, 1994), and was later confirmed by a large amount of experimental data (Haider et al., 2006; Okun and Lampl, 2008; Dorrn et al., 2010; Graupner and Reyes, 2013). Theoretical studies have found that the asynchronous irregular firing state spontaneously emerges in a network of excitatory and inhibitory neurons with random connections satisfying some very loose balancing conditions (van Vreeswijk and Sompolinsky, 1996; van Vreeswijk and Sompolinsky, 1998; Renart et al., 2010). The effects of this chaotic state on optimal coding (Denève and Machens, 2016), working memory (Lim and Goldman, 2014), and neuronal tuning (Hansel and van Vreeswijk, 2012), as well as its coexistence with attractor dynamics (Litwin-Kumar and Doiron, 2012) have been widely studied.

In the present study, we focus on the fast tracking ability of E-I balanced networks, where the population firing rate of the network is proportional to the input amplitude and tracks input changes rapidly (van Vreeswijk and Sompolinsky, 1996; Renart et al., 2010), and investigate how E-I balanced neural networks can be used for fast signal detection in brain-inspired computation. Neuromorphic computing, which mimics the structures and computational principles of the neural system, is receiving increasing attention in artificial intelligence (AI), as it has the potential to overcome the von Neumann bottleneck in modern computers that limits their processing speed (Indiveri and Liu, 2015). The fast response property of the E-I balanced network makes it a naturally compatible candidate to be implemented in neuromorphic systems to achieve rapid information processing.

In the following sections, we show that the asynchronous firing state of the network generates an optimal noise structure which enables the network to track input changes rapidly. We then extend the homogeneous connectivity of the classical E-I balanced neural network to include local neuronal connections, so that the network can achieve fast response and meanwhile maintain the spatial information when presented with spatially heterogeneous signals. Finally, we carry out simulations to demonstrate the performance of our model.

## 2. Fast Response of a Homogeneous E-I Balanced Network

To illustrate the mechanism of the fast response property, we first investigate a homogeneously connected E-I balanced network.

### 2.1. Intuition on the Mechanism of Fast Response

The fast response property of an E-I balanced network is at the population level. To understand this, let us consider a non-leaky linear integrate-and-fire neuron, whose dynamics is given by

(1) $$\begin{array}{l} \tau \frac{\text{d}v}{\text{d}t}=I, \end{array}$$

where τ is the integration time constant of the neuron, *v* the membrane potential, and *I* the input current. When *v* reaches the threshold θ, the neuron generates an action potential, and *v* is reset to the reset potential *v*_0_. Thus, for a constant input *I*_0_, the time it takes for a neuron to generate a spike starting from *v*_0_ is

$$\begin{array}{l} T=\tau \frac{\theta -v_{0}}{I_{0}}. \end{array}$$

**Figure 1:**
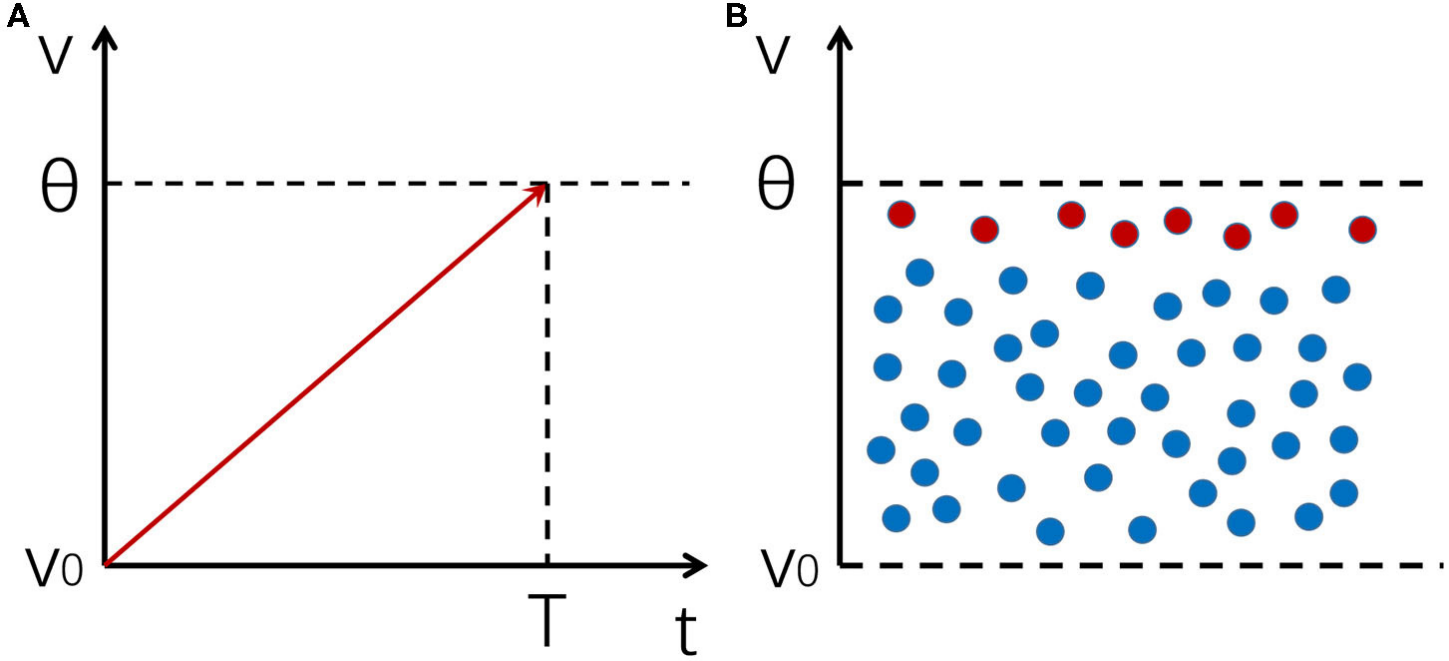
An illustration of the mechanism of fast response for a neural population. **(A)** The integration and firing process of a neuron receiving a noiseless input. The integration time is constrained by the membrane time constant. **(B)** A distribution of membrane potentials across a neural population enables it to respond to input changes rapidly. Red dots represent neurons whose potentials are close to the firing threshold, which are the first ones to respond to input changes.

It can be seen that the response time of a single neuron is limited by τ (Figure 1A).

However, when a neural population receives a signal, if the noise in the system keeps membrane potentials of different neurons at different levels, there will always be a few neurons whose potentials are near the threshold that can quickly respond to input changes. In such a case, the network as a whole can respond to input changes very fast, whose reaction time is only restricted by insurmountable factors such as axonal conduction delays, rather than the membrane time constant τ of individual neurons (Figure 1B). The key of this mechanism is to prevent synchronous firing of neurons and maintain a stable distribution of membrane potentials in the neural population, and asynchronous firing happens to be one of the hallmarks of an E-I balanced network (Renart et al., 2010), which we shall discuss in more detail below.

### 2.2. The Balancing Condition

We first present the conditions for maintaining an E-I balanced neural network and the stationary population firing rates under those conditions in the large *N* limit, where *N* is the number of neurons (van Vreeswijk and Sompolinsky, 1998; Rosenbaum et al., 2017). Consider a network of size *N*, with *N*_*E*_ = *q*_*E*_*N* being excitatory and *N*_*I*_ = *q*_*I*_*N* inhibitory, where *q*_*E*_ + *q*_*I*_ = 1. The input current received by neuron *i* in population *a* (*a* = *E* being excitatory and *a* = *I* being inhibitory) can be written as

(2) $$\begin{array}{l} I_{i}^{a}\left(t\right)=F_{i}^{a}\left(t\right)+R_{i}^{a}\left(t\right),\;a=E,I, \end{array}$$

where $F_{i}^{a}$ is the feedforward (i.e., external) input, and $R_{i}^{a}$ is the recurrent input from other neurons in the network with the form

(3) $$\begin{array}{l} R_{i}^{a}\left(t\right)=\underset{b=E,I}{\sum }\underset{j}{\sum }J_{ij}^{ab}\underset{k}{\sum }\frac{1}{\tau_{b,s}}e^{-\left(t-t_{j,k}\right)/\tau_{b,s}},\;a=E,I, \end{array}$$

where *j* indexes presynaptic neurons, τ_*b,s*_ is the synaptic time constant of the presynaptic population *b*, and *t*_*j,k*_ is the spike time of the *k*'th spike of neuron *j*.

Since in both the cortex and industrial applications, the number of neurons in a network is large, we may examine the balanced network in the *N* → ∞ limit. Expressing the relevant quantities in orders of *N* can help elucidate the mechanism. Neurons in the network are connected randomly, with the connection probability determined solely by the neuron types. The probability that neuron *j* in population *b* connects to neuron *i* in population *a* is *p*_*ab*_ for all *i, j*. Note that here *p*_*ab*_ is constant, and does not tend to 0 as *N* → ∞. This regime is usually referred to as dense connectivity, in contrast to sparse connectivity where the number of presynaptic neurons for each postsynaptic neuron is kept constant as *N* → ∞ (van Vreeswijk and Sompolinsky, 1996; Brunel, 2000). If a connection exists, its strength is set to be $J_{ij}^{ab}=j_{ab}/\sqrt{N}$; otherwise $J_{ij}^{ab}=0$. Here $j_{ab}\sim O\left(1\right)$. (O denotes scaling with respect to *N* → ∞ throughout this paper.) This scaling is a hallmark of balanced networks. Note that in some earlier works, especially those that employ a sparse connectivity regime (van Vreeswijk and Sompolinsky, 1996), this scaling is often written as $J\sim O\left(\sqrt{K_{ab}}\right)$, where *K*_*ab*_ is the average number of presynaptic inputs from population *b* for a neuron in population *a*. Here, since we have *K*_*ab*_ = *p*_*ab*_*N* and $p_{ab}\sim O\left(1\right)$, these two scalings are essentially the same.

Using the mean-field approximation, the time- and population-averaged input current received by a neuron in population *a* can be written as,

(4) $$\begin{array}{l} \bar{I_{a}}=\bar{F_{a}}+\bar{R_{a}}=\sqrt{N}\left(f_{a}\mu_{0}+w_{aE}r_{E}+w_{aI}r_{I}\right),\;a=E,I, \end{array}$$

where *r*_*b*_ is the mean firing rate of population *b*, *b* = *E, I*, and $w_{ab}=p_{ab}j_{ab}q_{b}\sim O\left(1\right)$. Here, we have written $\bar{F_{a}}$ as $\bar{F_{a}}=\sqrt{N}f_{a}\mu_{0}$, where $f_{a},\mu_{0}\sim O\left(1\right)$, because if we notice that long-distance projections are mainly excitatory, and assume that the feedforward inputs originated from another neural population of size O(N) and that the feedforward synaptic strength is also of order $O\left(1/\sqrt{N}\right)$, then $\bar{F_{a}}\sim O\left(\sqrt{N}\right)$ is a natural consequence. This is exactly the case in the Spike Camera data scenario that we shall examine later in section 3.

Therefore, to keep *I* (and thus *r*) bounded when *N* → ∞, we must have

$$\begin{array}{l} w_{aE}r_{E}+w_{aI}r_{I}+f_{a}\mu_{0}\sim O\left(\frac{1}{\sqrt{N}}\right),\;a=E,I. \end{array}$$

Letting *N* → ∞, we get approximate firing rates in the large *N* limit

(5) $$\begin{array}{l} \begin{array}{c} \underset{N\to \infty }{\lim}r_{E}=\frac{f_{E}w_{II}-f_{I}w_{EI}}{w_{EI}w_{IE}-w_{EE}w_{II}}\mu_{0},\\ \underset{N\to \infty }{\lim}r_{I}=\frac{f_{I}w_{EE}-f_{E}w_{IE}}{w_{EI}w_{IE}-w_{EE}w_{II}}\mu_{0}. \end{array} \end{array}$$

To keep the above limits positive and yield a stable solution, it is necessary and sufficient to let (van Vreeswijk and Sompolinsky, 1998)

$$\begin{array}{l} \frac{f_{E}}{f_{I}}>\frac{w_{EI}}{w_{II}}>\frac{w_{EE}}{w_{IE}}. \end{array}$$

This is the condition for the balanced firing state.

It is worth noting that whatever the neuronal transfer function is, the population firing rate in the large *N* limit is always linearly proportional to μ_0_. That is, Equation (5) always holds. This is a direct result of Equation (4), where the total input current is the linear sum of the three $O\left(\sqrt{N}\right)$ order terms. The balanced firing state is a stable solution dynamically formed by the network (van Vreeswijk and Sompolinsky, 1998; Renart et al., 2010), and therefore requires no fine tuning of parameters such as *j*_*ab*_, which is different from some other models that also try to recreate the asynchronous irregular firing state (e.g., Brunel, 2000).

It should be pointed out that Equation (5) only gives the O(1) order term of *r*_*a*_. To satisfy the specific transfer function of neurons while maintaining the balance of the O(1) order term, the firing rates are adjusted by an $O\left(1/\sqrt{N}\right)$ term, which results in a O(1) order correction to *I* (Equation 4). We will come back to this in the specific case presented in the next section.

### 2.3. The Mechanism of Fast Response

As previously mentioned, the asynchronous firing of neurons is the key for fast response of the network. When the balancing conditions presented in the previous section are met, the network can achieve asynchronous irregular firing (Renart et al., 2010). We next use a network of non-leaky linear integrate and fire neurons to study the mechanism of fast response in more detail. Notably, this simple neuron model has already been implemented in a neuromorphic system (Fusi and Mattia, 1999). While not biologically realistic, this model captures the key characteristics of integrate-and-fire neurons crucial for neuromorphic computing.

The neuronal dynamics is given by Equation (1). For simplicity, let *v*_0_ = 0. It can be easily seen that the transfer function of this neuron is threshold-linear, i.e.,

(6) $$\begin{array}{l} r=\{\begin{array}{cc} \frac{\bar{I}}{\theta \tau },\; & \bar{I}⩾0,\\ 0, & \bar{I}<0. \end{array} \end{array}$$

Substituting this into Equation (4) yields the population firing rates of excitatory and inhibitory neurons

(7) $$\begin{array}{l} \begin{array}{c} r_{E}=\frac{\left(f_{E}w_{II}-f_{I}w_{EI}\right)-\frac{1}{\sqrt{N}}f_{E}\theta \tau_{I}}{\left(w_{EI}w_{IE}-w_{EE}w_{II}\right)+\frac{1}{\sqrt{N}}\theta \left(w_{EE}\tau_{I}+w_{II}\tau_{E}\right)-\frac{1}{N}\theta^{2}\tau_{I}\tau_{E}}\mu_{0},\\ r_{I}=\frac{\left(f_{I}w_{EE}-f_{E}w_{IE}\right)-\frac{1}{\sqrt{N}}f_{I}\theta \tau_{E}}{\left(w_{EI}w_{IE}-w_{EE}w_{II}\right)+\frac{1}{\sqrt{N}}\theta \left(w_{EE}\tau_{I}+w_{II}\tau_{E}\right)-\frac{1}{N}\theta^{2}\tau_{I}\tau_{E}}\mu_{0}. \end{array} \end{array}$$

Comparing the above result with Equation (5), we can see that they are indeed $O\left(1/\sqrt{N}\right)$ order corrections to the *N* → ∞ limit, as stated at the end of the last section. Note that the firing rates still linearly encode the external input, which is a result of the threshold-linear transfer function. We also check that even when the external input is small or the number of neurons is not large, the linear encoding property still holds, which expands the dynamic range of the network. However, for other non-linear neuron models, this linear encoding property may not hold.

Equation (7) is derived from the mean-field approximation, that is, it is the result of averaging over time and neurons when the system reaches a stable state. To study how the instantaneous firing rate of the population changes with time when external input changes, we need more detailed analysis. We shall use the Fokker-Planck equation (Risken, 1996) to study the membrane potential distribution *p*_*a*_(*v, t*) (Brunel and Hakim, 1999; Fusi and Mattia, 1999; Brunel, 2000; Huang et al., 2011).

First, we examine the input received by a single neuron as described in Equation (2). We consider an external input signal with additive white Gaussian noise

(8) $$\begin{array}{l} F_{i}^{a}\left(t\right)=\sqrt{N}f_{a}\mu_{F}\left(t\right)+\sigma_{aF}\left(t\right)\xi_{i}^{aF}\left(t\right),\;a=E,I,\;i=1,\cdots \;,N_{a}, \end{array}$$

where $\xi_{i}^{aF}$ is a Gaussian white noise of magnitude 1 that is independent across neurons. Note that the signal mean is of order $O\left(\sqrt{N}\right)$, while the variance is of order O(1). This is because if we continue to use the settings considered before, and view the feedforward input as coming from Poisson spike trains generated by O(N) neurons firing at rates of order O(1), and transmitted through synapses with the strength of order $O\left(1/\sqrt{N}\right)$, then the resulting input's variance is the sum of O(N) number of terms with the same order as the square of synaptic strengths (O(1/N)), and is therefore of order O(1). This characteristic is also present in the later analysis of recurrent inputs.

Next, we examine the recurrent inputs. When the network enters the balanced state, since the neurons fire asynchronously (Renart et al., 2010), and the effect of each spike is small, we could use Gaussian white noise to approximate the variations of recurrent inputs, and rewrite the second term in Equation (2) as (Brunel, 2000)

(9) $$\begin{array}{l} R_{i}^{a}\left(t\right)=\sqrt{N}\mu_{aR}\left(t\right)+\sigma_{aR}\left(t\right)\xi_{i}^{aR}\left(t\right),\;a=E,I, \end{array}$$

where

(10) $$\begin{array}{l} \mu_{aR}=w_{aE}r_{E}+w_{aI}r_{I},\;\sigma_{aR}^{2}=j_{aE}w_{aE}r_{E}+j_{aI}w_{aI}r_{I}, \end{array}$$

and $\xi_{i}^{aR}$ is Gaussian noise of magnitude 1. The terms $\xi_{i}^{aR}$ and $\xi_{i}^{aF}$ are independent due to the asynchronous firing state, and can therefore be merged into one noise source. Thus, we transform Equation (2) into

(11) $$\begin{array}{l} I_{i}^{a}\left(t\right)=\mu_{a}\left(t\right)+\sigma_{a}\left(t\right)\xi_{i}^{a}\left(t\right),\;a=E,I, \end{array}$$

where

(12) $$\begin{array}{l} \mu_{a}=\sqrt{N}\left(w_{aE}r_{E}+w_{aI}r_{I}+f_{a}\mu_{F}\right)\\ \sigma_{a}^{2}=j_{aE}w_{aE}r_{E}+j_{aI}w_{aI}r_{I}+\sigma_{aF}^{2}, \end{array}$$

and $\xi_{i}^{a}$ is Gaussian white noise of magnitude 1. Also note that the mean of the signal is consistent with Equation (4), and the mean and variance are both of order O(1).

Since the balanced state implies asynchronous firing (Renart et al., 2010), the noise $\xi_{i}^{a}$ of different neurons can be seen as independent. Then, the excitatory (inhibitory) population can be viewed as i.i.d. samples of the same random process. The membrane potential distribution of population *a*, *p*_*a*_(*v, t*), can thus be derived from Equation (11). We obtain the Fokker-Planck equation (Brunel, 2000; Huang et al., 2011)

(13) $$\begin{array}{l} \tau_{a}\frac{\partial p_{a}\left(v,t\right)}{\partial t}=-\mu_{a}\frac{\partial p_{a}\left(v,t\right)}{\partial v}+\frac{\sigma_{a}^{2}}{2\tau_{a}}\frac{\partial^{2}p_{a}\left(v,t\right)}{\partial v^{2}},\;a=E,I. \end{array}$$

A few boundary conditions can be naturally imposed (Brunel and Hakim, 1999; Brunel, 2000):

(14) $$\begin{array}{l} p_{a}\left(v,t\right)=0,\;\forall v⩾\theta . \end{array}$$

(15) $$\begin{array}{l} p_{a}\left(0^{-},t\right)=p_{a}\left(0^{+},t\right), \end{array}$$

(16) $$\begin{array}{l} \frac{\partial p_{a}\left(0^{+},t\right)}{\partial v}-\frac{\partial p_{a}\left(0^{-},t\right)}{\partial v}=\frac{\partial p_{a}\left(\theta ,t\right)}{\partial v}. \end{array}$$

(17) $$\begin{array}{l} \int_{-\infty }^{\theta }p_{a}\left(v,t\right)\text{d}v=1. \end{array}$$

**Figure 2:**
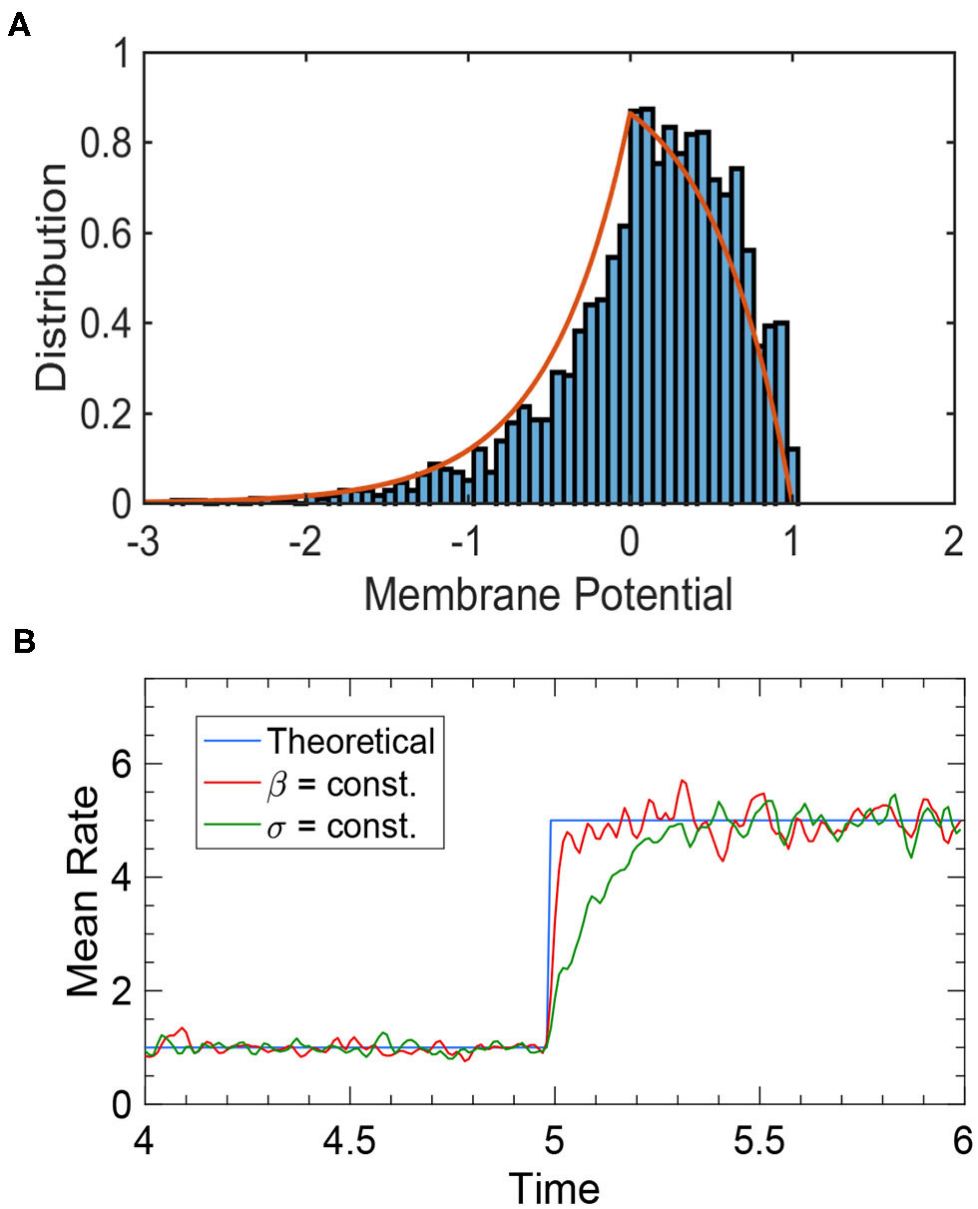
Simulation results of an uncoupled neural population. **(A)** The membrane potential distribution of a neural population receiving independent white noise-corrupted signals with a constant VMR of 1. The red curve is the theoretical prediction given by Equation (18), and the blue histogram is the actual simulation result. **(B)** The tracking performance of a neural population depends on the input noise structure. The blue curve is the theoretical prediction of steady-state firing rate given by Equation (19). The red curve is the network performance when the VMR is constant (β = 1), which tracks the input change almost instantaneously. The green curve is the network performance when the noise variance, rather than the VMR, is constant (σ ≡ 1), where a significant delay is present. Other parameters are: *N* = 2, 500, τ = 1, θ = 1, and μ changing from 1 to 5 at time *t* = 5.

In Equation (13), letting ∂*p*_*a*_/∂*t* = 0, and using the above boundary conditions, we get the stationary solution

(18) $$\begin{array}{l} p_{a0}\left(v\right)=\{\begin{array}{cc} \frac{1}{\theta }\left[1-\exp\left(-2\tau_{a}\beta_{a}\right)\right]\exp\left(\frac{2\tau_{a}v}{\beta_{a}}\right), & v<0\\ \frac{1}{\theta }\left[1-\exp\left(\frac{-2\tau_{a}\left(\theta -v\right)}{\beta_{a}}\right)\right], & 0⩽v⩽\theta \\ 0, & v>\theta \end{array} \end{array}$$

where $\beta_{a}: =\sigma_{a}^{2}/\mu_{a}$ is the variance-to-mean ratio (VMR). This result is confirmed by simulations (Figure 2A).

The population firing rate, i.e., the flux at θ, is

(19) $$\begin{array}{l} {r_{a}=-\frac{\sigma_{a}^{2}}{2\tau_{a}^{2}}\frac{\partial p_{a0}(v)}{\partial v}|}_{\theta }=\frac{\mu_{a}}{\theta \tau_{a}}. \end{array}$$

which is consistent with Equation (6).

It can be seen from Equation (18) that the membrane potential distribution is determined by the VMR β_*a*_. The ideal noise structure is thus obtained when VMR stays constant (Huang et al., 2011), because it ensures that when the external input μ_*F*_ changes, the system remains in a stationary state where Equation (19), and thus Equation (7), always holds. In this way, the population rate can track input changes instantaneously and linearly encode μ_*F*_ at all times. Figure 2B illustrates how the response time of the population rate is determined by input noise structure.

From Equations (12) and (19), we know that when the network is at the stationary state,

$$\begin{array}{l} \beta_{a}=\frac{\sigma_{a}^{2}}{\mu_{a}}=\frac{j_{aE}w_{aE}r_{E}+j_{aI}w_{aI}r_{I}+\sigma_{aF}^{2}}{\theta \tau_{a}r_{a}},\;a=E,I. \end{array}$$

**Figure 3:**
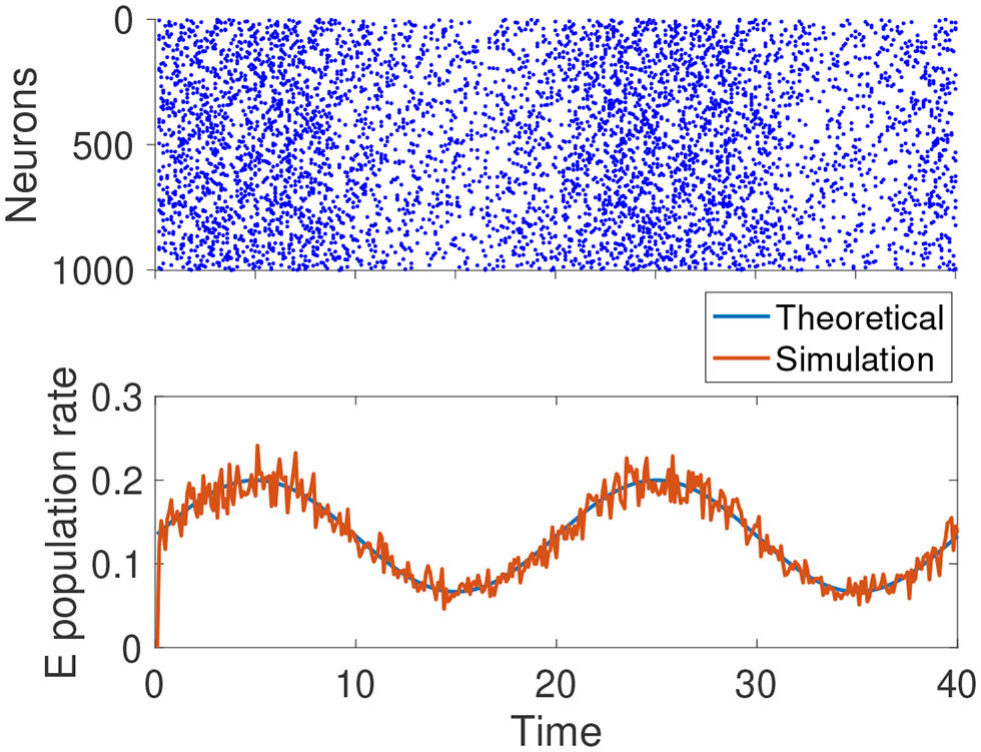
Simulation results of a homogeneous E-I balanced network tracking a time-varying input. The network receives a sinusoidal input centered at μ_*F*_ = 0.1 with an amplitude of 0.05. $\sigma_{aF}^{2}/\mu_{F}=0.1$ remains constant. The blue curve is the theoretic prediction given by Equation (7). The red curve is the instantaneous average firing rate of excitatory neurons. The parameters are: $N=1\times 10^{4},q_{I}=0.2,p_{ab}=0.25,\theta =15,\tau_{E}=15,\tau_{I}=10,\tau_{E,s}=6,\tau_{I,s}=5,f_{E}=3,f_{I}=2,j_{EE}=0.25,j_{EI}=-1,j_{IE}=0.4,j_{II}=-1$.

From Equation (7), we know *r*_*E*_, *r*_*I*_ ∝ μ_*F*_. For the $\sigma_{aF}^{2}$ term, if we continue to assume that the external input comes from the Poisson spike trains of another population of neurons, and the changes in μ_*F*_ are due to the firing rate of that population, then we have $\sigma_{aF}^{2}\propto \mu_{F}$. Thus, when μ_*aF*_ changes, β_*a*_ remains constant. This is the ideal noise structure, and the population rate of the network can track the external input instantaneously. In reality, the ideal noise structure can only be approximately satisfied, but the tracking speed of the network is still reasonably fast, as confirmed by Figure 3.

It should be pointed out that the neuron model we used in this section does not have a lower bound to its membrane potential. In real applications, a reflecting barrier can be imposed at the reset potential *v*_0_ (Fusi and Mattia, 1999). We verify that this does not affect our main results. The neuron model used in the following sections has a reflecting barrier.

## 3. Processing Spatially Heterogeneous Input With Local Connectivity

In the above, we have studied an E-I balanced neural network with homogeneous connectivity, which is able to track input changes rapidly. However, when the external input is spatially heterogeneous, that is, when different neurons receive inputs of different magnitudes, this homogeneous connectivity generates statistically equivalent recurrent inputs for each neuron that cannot balance the external inputs. The same $R_{a}^{i}$'s cannot balance different $F_{a}^{i}$'s, causing neurons to receive inputs of order $O\left(\sqrt{N}\right)$ and fire pathologically. In addition, the random long-range connections between neurons spread out local activities to the entire network, which blurs the spatial location of inputs. In applications, however, we often need to know not only when the signal occurs but also where it occurs. To solve this problem, we need to introduce local connectivity in the network. Previous studies have shown that if appropriate local connectivity is included, the network can maintain the balanced firing state as well as retain the spatial information of the input (Rosenbaum and Doiron, 2014; Rosenbaum et al., 2017), which enables the network to achieve both fast tracking and spatial location encoding. Below, we briefly introduce the balancing conditions and the response property of an E-I balanced neural network with local connectivity.

Here, each neuron is assigned a location (*x, y*) on the plane, and local connectivity is achieved by a connection probability that decays with the spatial distance between pairs of neurons instead of being homogeneous as in the previous sections, so that neurons closer to each other have higher probabilities to connect with each other. Specifically, the probability of a connection between neurons *i* and *j* follows

(20) $$\begin{array}{l} \mathbb{P}\left(j\text{ connects to }i\right)\propto G_{b}\left(d_{ij}\right), \end{array}$$

where *G*_*b*_ is a 2-dimensional Gaussian shaped function whose spatial spread is determined by the presynaptic population *b*, and *d*_*ij*_ is the distance between the neurons.

Similar to Equation (4), we again utilize the mean-field approximation. Only this time, we do not average over the entire population, but rather approximate the neural activity of population *a* near location **x** with the neural field

(21) $$\begin{array}{l} \bar{I_{a}}\left(\text{x}\right)=\bar{F_{a}}\left(\text{x}\right)+\bar{R_{a}}\left(\text{x}\right)=\sqrt{N}\left[f_{a}\left(\text{x}\right)+w_{aE}*r_{E}\left(\text{x}\right)-w_{aI}*r_{I}\left(\text{x}\right)\right],\\ \;a=E,I, \end{array}$$

where the feedforward input ${\bar{F}}_{a}\left(\text{x}\right)=\sqrt{N}f_{a}\left(\text{x}\right)$, *w*_*ab*_(**x**) = *q*_*b*_*j*_*ab*_*p*_*ab*_*G*_*b*_(**x**) is the mean connectivity a neuron in population *a* receives from neurons in population *b* at location **x**, and *r*_*a*_(**x**) is the firing rate. The symbol * denotes the spatial convolution against **x**.

Similar to section 2.2, we have

(22) $$\begin{array}{l} w_{aE}*r_{E}\left(\text{x}\right)-w_{aI}*r_{I}\left(\text{x}\right)+f_{a}\left(\text{x}\right)\sim O\left(1/\sqrt{N}\right),\;a=E,I. \end{array}$$

Let *N* → ∞ and perform 2-dimensional Fourier transform against **x**, and we get

$$\begin{array}{l} {\tilde{w}}_{aE}{\tilde{r}}_{E}-{\tilde{w}}_{aI}{\tilde{r}}_{I}+{\tilde{f}}_{a}=0,\;a=E,I, \end{array}$$

where the symbol ˜ denotes the spatial Fourier transform. This gives

(23) $$\begin{array}{l} {\tilde{r}}_{E}=\frac{{\tilde{f}}_{E}{\tilde{w}}_{II}-{\tilde{f}}_{I}{\tilde{w}}_{EI}}{{\tilde{w}}_{EI}{\tilde{w}}_{IE}-{\tilde{w}}_{EE}{\tilde{w}}_{II}},\;{\tilde{r}}_{I}=\frac{{\tilde{f}}_{E}{\tilde{w}}_{IE}-{\tilde{f}}_{I}{\tilde{w}}_{EE}}{{\tilde{w}}_{EI}{\tilde{w}}_{IE}-{\tilde{w}}_{EE}{\tilde{w}}_{II}}. \end{array}$$

To ensure that the above Fourier transform exists, it is necessary that ${\tilde{r}}_{a}$ tends to 0 as the frequency tends to infinity. This requires that the external input *f* be “wider” than recurrent input *w*. This can be understood intuitively from Equation (22), where we see convolution makes *w*_ab_*r_*b*_(**x**) wider than *w*_*ab*_(**x**), so for the terms to balance each other, *f* has to be “wider” than *w*. Also, to get a positive stable solution, the following condition has to be met:

(24) $$\begin{array}{l} \frac{{\bar{f}}_{E}}{{\bar{f}}_{I}}>\frac{{\bar{w}}_{EI}}{{\bar{w}}_{II}}>\frac{{\bar{w}}_{EE}}{{\bar{w}}_{IE}}, \end{array}$$

where the bar represents spatial average. Also, to make the solution stable, *w*_*aE*_ has to be “wider” than *w*_*aI*_. For a more detailed account of these conditions, see Rosenbaum and Doiron, 2014; Pyle and Rosenbaum, 2017.

Rosenbaum et al. (2017) proved the asynchronous firing state of the network with local connections under the above conditions. Thus, with the premise of asynchronous firing satisfied, our results regarding the optimum noise structure in section 2.3 still holds. Let the total input variance of the neuron in population *a* at location **x** be $\sigma_{a}^{2}\left(\text{x}\right)$, and the VMR be β_*a*_(**x**), and we have

$$\begin{array}{l} \sigma_{a}^{2}\left(\text{x}\right)=j_{aE}w_{aE}*r_{E}\left(\text{x}\right)+j_{aI}w_{aI}*r_{I}\left(\text{x}\right). \end{array}$$

The threshold-linear transfer function gives us $\bar{I_{a}}\left(\text{x}\right)=\theta \tau_{a}r_{a}\left(\text{x}\right)$, so we have

$$\begin{array}{l} \beta_{a}\left(\text{x}\right)=\frac{j_{aE}w_{aE}*r_{E}\left(\text{x}\right)+j_{aI}w_{aI}*r_{I}\left(\text{x}\right)}{\theta \tau_{a}r_{a}\left(\text{x}\right)}, \end{array}$$

Here the division is point-wise at each **x**. If β_*a*_(**x**) is constant at each **x** for arbitrary external input *f*_*a*_(**x**), it must be spatially invariant, that is, β_*a*_(**x**) ≡ β_*a*_. We can thus move the denominator on the r.h.s. to the left, and perform Fourier transform to get

$$\begin{array}{l} \beta_{a}\theta \tau_{a}{\tilde{r}}_{a}=j_{aE}{\tilde{w}}_{aE}*{\tilde{r}}_{E}\left(\text{x}\right)+j_{aI}{\tilde{w}}_{aI}*{\tilde{w}}_{I}\left(\text{x}\right),\;a=E,I. \end{array}$$

Substituting it in Equation (23), we get

$$\begin{array}{l} \begin{array}{c} -\frac{j_{EE}{\tilde{w}}_{EE}}{j_{EI}{\tilde{w}}_{EI}}=\frac{{\tilde{f}}_{E}{\tilde{w}}_{IE}-{\tilde{f}}_{I}{\tilde{w}}_{EE}}{{\tilde{f}}_{E}{\tilde{w}}_{II}-{\tilde{f}}_{I}{\tilde{w}}_{EI}},\\ -\frac{j_{II}{\tilde{w}}_{II}}{j_{IE}{\tilde{w}}_{IE}}=\frac{{\tilde{f}}_{E}{\tilde{w}}_{II}-{\tilde{f}}_{I}{\tilde{w}}_{EI}}{{\tilde{f}}_{E}{\tilde{w}}_{IE}-{\tilde{f}}_{I}{\tilde{w}}_{EE}}. \end{array} \end{array}$$

The above equations cannot be satisfied for all *f*_*a*_, so this network structure cannot maintain an optimum noise structure and track any input instantly. However, for input changes that only concerns magnitude and not the spatial shape, β_*a*_(**x**) can remain constant and allow instant tracking. For other kinds of input changes, although instantaneous tracking is not possible, the response speed of the network is still significantly smaller than what the neuronal time constant allows, as we shall explore in the next section.

## 4. Simulation Results

One of the potential applications of the balanced network's fast response property is to process Spike Camera data in real time. Spike Camera is a newly developed neuromorphic hardware that encodes visual signals with spikes (Dong et al., 2017). It consists of artificial ganglion cells, each corresponding to a pixel, that linearly integrate the luminance intensity and fire a spike upon reaching the threshold, converting continuous visual information to discrete spikes. This event-based data transmission method significantly reduces the data volume and allows for a sampling rate of as high as 40,000 fps. Compared to another extremely high-speed camera, the Dynamic Vision Sensor (DVS) (Serrano-Gotarredona and Linares-Barranco, 2013), which only transmits changes in light intensity, Spike Camera can directly encode the absolute value of the luminance signal with its spiking rate while having an even higher sampling rate. In this section, we explore the tracking performance of our network under the setting of processing Spike Camera-like data.

### 4.1. Network Structure

**Figure 4:**
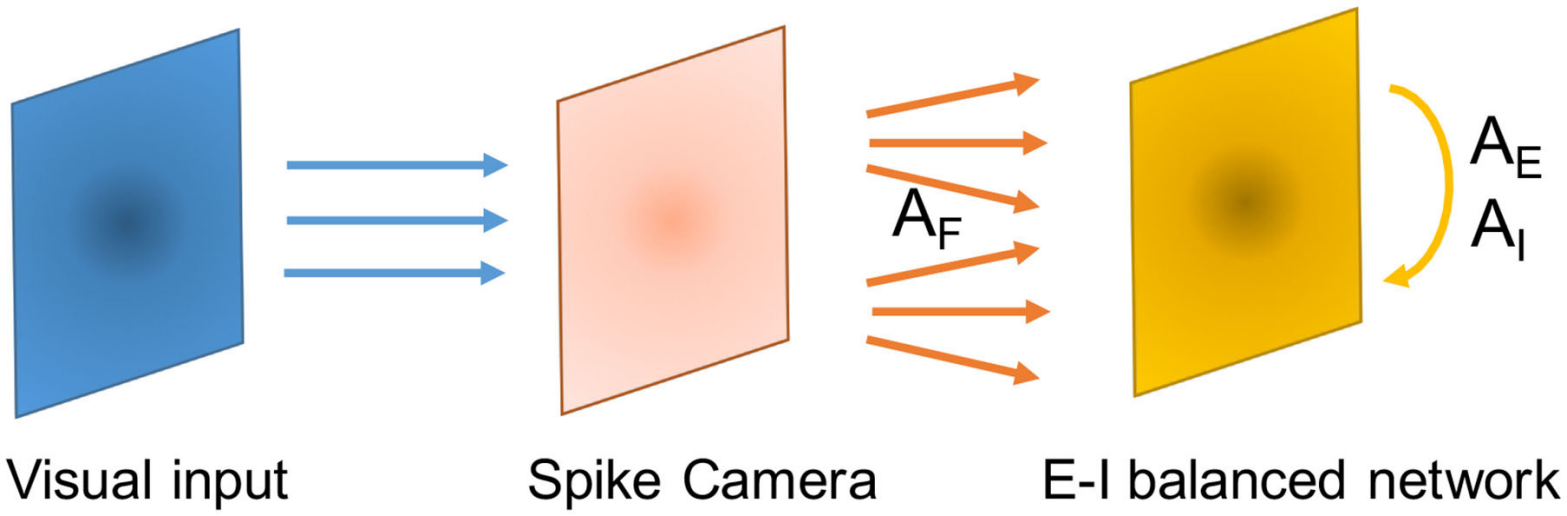
Schematic of the network structure in the context of processing Spike Camera data.

We use a feedforward layer consisting of 50 × 50 non-leaky linear integrate-and-fire neurons to mimic the Spike Camera. Each neuron in this layer receives visual signal from its corresponding pixel location, and connects to the balanced network layer through feedforward connections $J_{ij}^{aF},a=E,I$. The balanced network layer consists of 80 × 80 excitatory neurons and 40 × 40 inhibitory neurons. The neurons of each population is placed uniformly on a square area with a side length of 1. The neurons in the feedforward layer obeys Equation (1), and have a neuronal time constant of τ_*F*_. To reflect the high sampling rate of Spike Camera, τ_*F*_ is set to be very small. The connection probability of the network obeys

$$\begin{array}{l} \mathbb{P}\left(J_{ij}^{ab}=j_{ab}/\sqrt{N}\right)=p_{ab}G_{b}\left(d_{ij}^{ab}\right),\;b=F,E,I,\;a=E,I, \end{array}$$

where *F* stands for the feedforward layer, *G*_*b*_ is a 2-dimensional Gaussian distribution centered at 0 with scale parameter *A*_*b*_. To satisfy the balancing conditions, we let *A*_*F*_ > *A*_*E*_ ⩾ *A*_*I*_ and make sure that Equation (24) holds. Since spatial location is discretized in the network, to keep the total connection probability from population *b* to population *a* at *p*_*ab*_, we normalize *G*_*b*_ by letting $\sum_{i}G_{b}\left(d_{ij}^{ab}\right)=1,\forall j$. Figure 4 demonstrates this structure.

### 4.2. Tracking Time-Varying Stimuli

**Figure 5:**
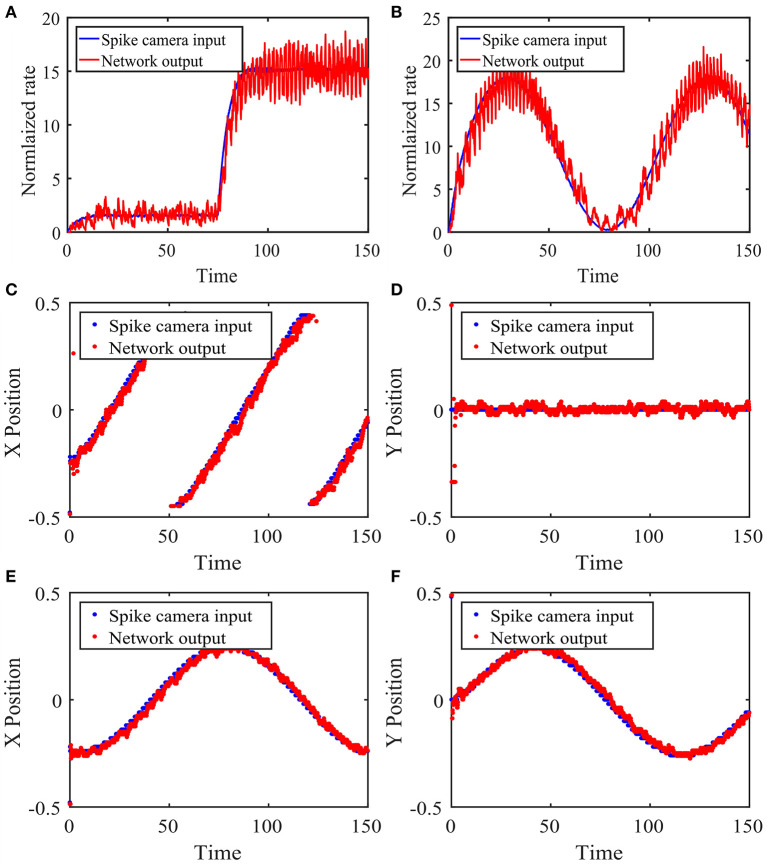
Performance of the network with local connections in response to time-varying stimuli. **(A)** Network response to the sudden appearance of an object. The Spike Camera layer receives a disc-shaped visual input centered at (0.25, 0.5) with a radius of 0.05, whose magnitude changes abruptly from 1.5 to 15 at *t* = 75. A background noise is added. The blue curve is the firing rate of the area corresponding to the visual input in the Spike Camera layer. The red curve is the rate of the excitatory neurons at the same area in the balanced network layer, which is normalized for better comparison with the blue curve. **(B)** Same as panel **(A)**, except that the input amplitude follows the sinusoidal function μ(*t*) = *A*(sin(B*2π*t*/*T*)) + *C, A* = 30, *B* = 3/2, *C* = 30. **(C,D)** The stimulus is an object moving across the visual field in constant velocity. The object has the same shape as panels **(A,B)**, with a magnitude of 10. Panels **(C,D)** show the tracking of the x and y coordinates, respectively. The blue curve is the object location decoded from the activity of the Spike Camera layer, and the red curve is that of the balanced network layer. **(E,F)** Same as panels **(C,D)**, except that the stimulus moves counterclockwise on a circle in constant speed. The network parameters are θ = 15, τ_*F*_ = 1, τ_*E*_ = 15, τ_*I*_ = 10, τ_*F,s*_ = τ_*E,s*_ = 5, τ_*I,s*_ = 2.5, *p*_*EF*_ = 0.05, *p*_*IF*_ = 0.025, *p*_*EE*_ = 0.02, *p*_*EI*_ = 0.08, *p*_*IE*_ = 0.06, *p*_*II*_ = 0.08, *A*_*F*_ = 0.05, *A*_*E*_ = 0.02, *A*_*I*_ = 0.02, *j*_*EF*_ = 140, *j*_*IF*_ = 93.3, *j*_*EE*_ = 80, *j*_*EI*_ = −320, *j*_*IE*_ = 40, *j*_*II*_ = −320.

We test the tracking performance of our network with four example input stimuli. The first stimulus is the sudden appearance of an object, modeled as an abrupt change in input magnitude at the object's location. Figure 5A shows the network's response to this change summarized by the population rate of the excitatory neurons corresponding to the location of interest. We see that in this case, the network's activity tracks the stimulus change very quickly.

The second stimulus is similar to the previous one, except that the input magnitude continuously changes in a sinusoidal manner. Figure 5B shows the tracking performance of the network. It can be seen that the network can track the stimulus almost instantaneously, which is expected since β_*E*_ is constant here.

The third stimulus is an object moving quickly from left to right in the field of vision, which can be seen as a model of a typical motion tracking task. We use the coordinates of the center of the circular object to represent the location of the stimulus. The coordinates calculated from the Spike Camera data and the balanced network activity are then compared in Figures 5C,D. The network activity closely tracks the input, and the spatial information is preserved.

The last stimulus is similar to the previous one, except that the motion is circular instead of linear, which implies a constantly changing velocity. The same method is used to locate the stimulus, and the results are shown in Figures 5E,F. The performance is again very good.

### 4.3. Trackable Speeds

**Figure 6:**
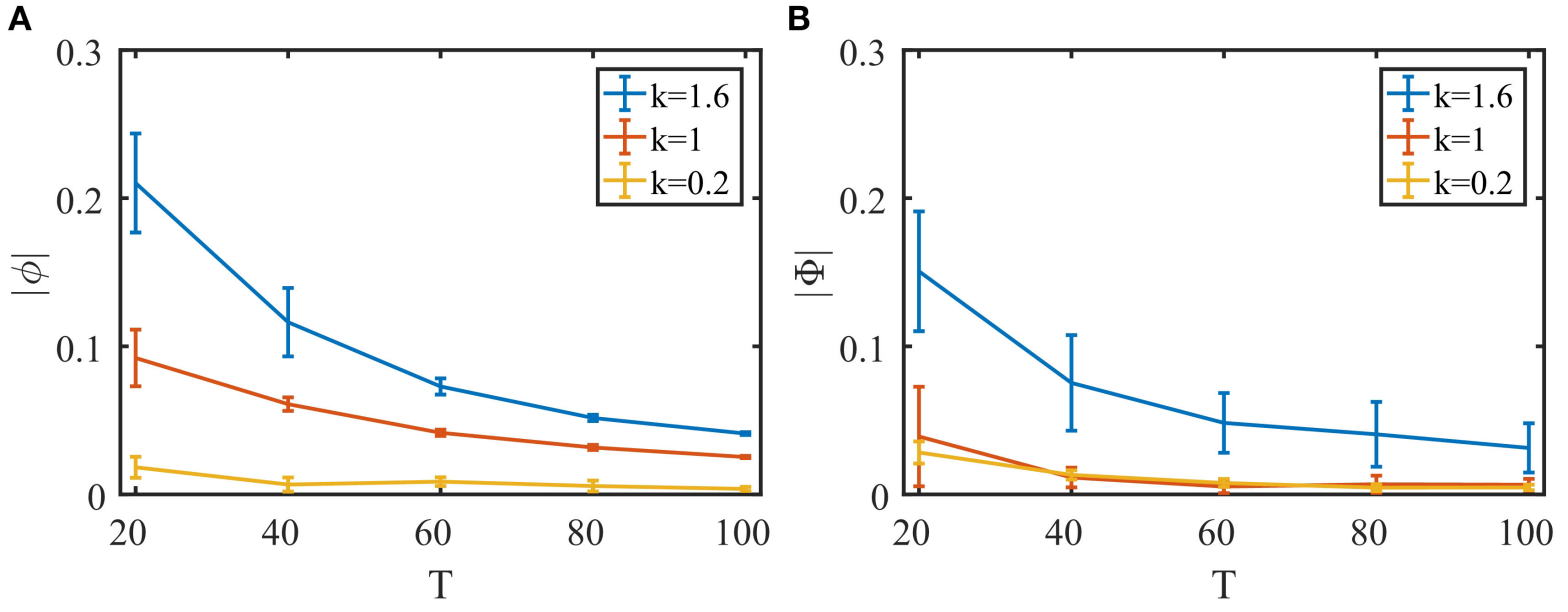
Quantifying the network's performance with temporal and spatial phase lag. To examine the effect of the synaptic time constant on tracking performance, we define τ_*b,s*_ = *kτ*_*b, s*0_, *b* = *F, E, I*, where τ_*b, s*0_ is the set of parameters used in Figure 5. **(A)** Temporal phase lag in the second task (Figure 5B) with different signal periods. **(B)** Spatial phase lag in the fourth task (Figures 5E,F) with different circular motion periods. Ten trials for each data point. Error bars show standard deviations.

To explore the extent of the network's tracking ability, we next evaluate the temporal and spatial lags of the response. We first change the frequency of the sinusoidal signal in the second task in the previous section (Figure 5B) and calculate the phase lag of the balanced network's response. As can be seen in Figure 6A, while the phase lag |ϕ| increases when the signal frequency 1/*T* is higher, the delay is still very small overall.

Next, we vary the speed of the object's circular motion in the fourth task in the previous section (Figures 5E,F) and evaluate the spatial phase lag of the object location decoded from the balanced network activity compared to that of the Spike Camera layer. As shown in Figure 6B, the tracking error is small even when the object is moving very quickly.

Since the encoding happens at the population level, input changes have to be propagated through the population to be successfully tracked, and this process is mediated by synaptic interactions. This lead us to suspect that the synaptic time constants τ_*b,s*_ could be a limiting factor for tracking performance. To study this, we varied τ_*b,s*_ in both the temporal and spatial tracking tasks. Indeed, as can be seen in Figure 6, a shorter synaptic time constant leads to better performance. In practice, the shape of the synaptic current can be designed to have a τ_*b,s*_ as small as possible. The real constricting factor is the synaptic transmission delay, which corresponds to the communication speed of the hardware, but this is expected to be insignificant given the highly compact nature of neuromorphic chips.

## 5. Discussion and Conclusion

This paper proposed an algorithm for fast response in neuromorphic systems based on E-I balanced networks, systematically analyzed its fast response mechanism, and introduced local connections to maintain balance and retain spatial information in the face of spatially heterogeneous inputs. Simulations verified that the network indeed performs well with rapidly changing input stimuli.

There are still some questions left to explore. For instance, we have mentioned that the network cannot keep an optimal noise structure at all times, and thus the membrane potential distribution will change with the input. A study of the transient dynamics during such changes could help us further improve the network performance. As another example, notice that most of the theoretical analyses in the paper were conducted in the limit of *N* → ∞. In real-world applications, we often have to track small objects, during which the number of neurons encoding it usually does not exceed a few hundred. Studying the finite-size effect could help us better understand the network dynamics.

Although we mainly discussed the case where the input comes from Spike Camera, the network structure we proposed is not limited to processing visual signal. The “location” of neurons can also correspond to tuning to different variables or representation of abstract features. To achieve real-time processing of high-frequency data, the fast response property is required for each computational process. There has been a lot of research discussing how to implement various computations on top of a balanced network (Barrett, 2012; Hansel and van Vreeswijk, 2012; Litwin-Kumar and Doiron, 2012; Lim and Goldman, 2014; Denève and Machens, 2016; Pyle and Rosenbaum, 2017). The asynchronous irregular state can be taken as a model of the spontaneous state in the cortex. With the spontaneous state as a global attractor, and the specific computations and memories as input-sensitive local attractors (Amit and Brunel, 1997; Litwin-Kumar and Doiron, 2012), the chaos in the network's balanced firing state can allow it to respond to specific inputs very rapidly and initiate the required computation. Besides the fast response property, the balanced state also has other computational advantages such as stochastic resonance (Barrett, 2012).

Neuromorphic computing systems colocalize computation and memory by mimicking neural structures like neurons and synapses. This allows it to circumvent the von Neumann bottleneck, granting it enormous potentials in processing speed (Indiveri and Liu, 2015). There has been a lot of work investigating possible mechanisms for fast neural response (e.g., Bharioke and Chklovskii, 2015; Yu et al., 2015) which could potentially complement the processing speed of neuromorphic systems, and the balance of excitation and inhibition we explored here is one of them. The model we proposed here, with its simple neuron model and connectivity structure, can be readily implemented in hardware and serve as a fast-responding module integrated in a general neuromorphic system for rapid information processing. This paper thus lays the groundwork for realizing various kinds of fast computation using balanced networks, especially in neuromorphic systems.

## Data Availability Statement

The raw data supporting the conclusions of this article will be made available by the authors, without undue reservation.

## Author Contributions

SW designed the project. GT, SL, and SW wrote the paper. GT did the theoretical analyses. GT, SL, and SW carried out simulations and data analysis. TH contributed important ideas.

## Conflict of Interest

The authors declare that the research was conducted in the absence of any commercial or financial relationships that could be construed as a potential conflict of interest.
